# A cross-national study on mental health, psychological distress and suicidal ideation among veterinarians in multiple European countries

**DOI:** 10.3389/fvets.2025.1634139

**Published:** 2025-09-15

**Authors:** Marietta Máté, Claire Helen Várnai, László Ózsvári

**Affiliations:** Department of Veterinary Forensics and Economics, University of Veterinary Medicine Budapest, Budapest, Hungary

**Keywords:** veterinary, mental health, psychological distress, burnout, suicidal ideation, work-related stressors

## Abstract

**Background:**

The suicide rate among veterinarians is alarmingly high, being twice that of other medical professionals and four times that of the general population. This study examined the occurrence of suicidal thoughts, tendencies, and mental health challenges among veterinarians in Hungary, Finland, Sweden, Germany, and from other Northern-European countries (Norway, Denmark and Estonia).

**Methods:**

An online questionnaire of 55 items was developed and distributed between July 2021 and February 2022. A total of 724 veterinarians participated: 236 from Hungary, 218 from Finland, 157 from Sweden, 77 from Germany, 26 from Estonia, 5 each from Denmark and Norway. Factors such as country, age, gender, weekly working hours, job position and length of annual leave were assessed. Data were analyzed using Pearson’s chi-square test.

**Results:**

The results showed that work-related stressors significantly contributed to negative mental health across all surveyed countries. Among these stressors, clients’ expectations for prompt diagnosis were rated particularly high, with a mean of 4.34 ± 0.84 points on a Likert scale of 1 to 5, especially in Germany and Hungary, a difference considered statistically significant (ANOVA: *p* < 0.0001). Many veterinarians also reported high levels of anxiety related to their conscientiousness and punctuality at work, particularly in Hungary. In contrast, the emotional impact of performing euthanasia was ranked low, although this varied significantly by country (ANOVA: *p* < 0.0001). Younger respondents (aged 23–34 years) more often reported negative effects of their work on their mental health. Female veterinarians were more likely to seek professional counseling compared to men. Older veterinarians and those in leadership positions were more likely to manage their mental health effectively and seek help when needed, compared to younger veterinarians.

**Conclusion:**

These results highlight a worrying pattern of emotional distress in the veterinary field. Although the underlying causes of suicide are complex and multifactorial, addressing specific occupational stressors can play a critical role in improving mental health and reducing psychological risk in the profession. This study contributes to the growing research by providing cross-national data from underrepresented Central and Northern European countries, underscoring the importance of mental health strategies tailored to cultural and systemic contexts in the veterinary profession.

## 1 Introduction

The World Health Organization (WHO) has estimated that more than 720,000 people die by suicide worldwide each year, which means that suicides account for around 1.1% of all deaths (1). Certain occupations, those in medically related fields, have been linked to an increased risk of suicide (2). Among these, veterinarians face a particularly high risk, with suicide rates double those of other medical professions and four times that of the general population (3–6).

Nett et al. found that veterinarians are more likely to experience serious psychological distress, have a history of depression, and have contemplated suicide compared to the general population. They are also 2.1 to 3.5 times more likely to commit suicide (7). Furthermore, a survey revealed that 89.1% of veterinarians considered suicide to be one of the most critical issues facing their profession, and 92.0% identified stress as a significant concern. In terms of future thinking and reflecting on the profession more than half of veterinarians would not recommend the profession (8). Low self-compassion is associated with higher levels of burnout, depression, and stress among veterinarians. In Spain, self-criticism and limited emotional self-help contributed significantly to the deterioration of professionals’ mental health (9).

International studies further underscore the gravity of the situation. According to a 2022 US study, 7% of veterinarians experienced suicidal thoughts in the past 12 months, although the rate of suicide attempts was lower (0.022%) (10). In another American study, 17.3% of veterinarians surveyed reported that they had considered suicide in the past 12 months (11). A Canadian study found that 28% of UK veterinarians had considered suicide in the past year, compared to 17% of Canadian veterinarians, which, while lower than the UK figure, is still higher than the Canadian national average of 12% (12). In Australia, the combined suicide rate in Western Australia and Victoria was 45.2 per 100,000, significantly higher than the national average of 11.8 per 100,000, with veterinarians having a suicide rate 3.8 times higher than the general population (13). According to a cross-sectional study conducted in Hong Kong, the risk of suicide among local veterinarians was 22.0% (14).

In Germany, Schwerdtfeger et al. reported that 19% of veterinarians had experienced suicidal thoughts in the past 2 weeks, compared to 5.7% in the general population. Their findings also indicated that veterinarians are six to seven times more likely to be at increased suicide risk than the general population (5). Finland, which had one of the highest suicide rates globally in 1992 (28.8 per 100,000) (15), has shown improvements over time. However, between 2001 and 2005, veterinary medicine ranked as the third-highest profession for suicide among women. Nevertheless, this was a considerable improvement from a study performed between 1979 and 1982, which showed that veterinarians had the highest rate of suicide per occupation among both men and women (16). In Norway, census data from 1960 to 1990 showed that male veterinarians had nearly double the suicide rate of the general population (17). According to a Portuguese online survey, among veterinary professionals, 3.5% reported having attempted suicide during their lifetime, while 17.2% experienced extremely severe depression and suicidal ideation (18). Most of the studies (5, 10–12, 14, 18) were based on self-selected convenience samples, and therefore their findings reflect the experiences of participating veterinarians rather than occurrence rates in the broader veterinary population.

Interestingly, the suicide rates among veterinarians varied across Northern European countries. In Denmark, the highest suicide rates were observed among doctors and nurses rather than veterinarians (2, 19). Hawtom et al. found that from 1981 to 2006, suicide rates in Denmark were higher among physicians, dentists, nurses, and pharmacists than among veterinarians (19).

The decision to take one’s own life is influenced by a complex interplay of factors. The common stimulus in suicide is intolerable psychological pain, with hopelessness and/or helplessness as prevalent emotions, and a cognitive state of constriction. While mental health conditions are a significant risk factor, suicide is often the outcome of a combination of personal and situational factors (20).

In the veterinary profession, several occupational stressors have been identified as potential contributors to suicide risk. These include long working hours, heavy workloads, financial pressures, work-life balance challenges, lack of professional support, job satisfaction and emotionally demanding tasks such as performing euthanasia, delivering bad news, and meeting client expectations (3, 21–24). In addition, veterinarians may have easier access to lethal means such as euthanasia drugs or firearms, which is considered a profession-specific risk factor (25). Bartram et al. found that veterinary surgeons experience higher levels of anxiety and depressive symptoms, greater incidence of suicidal thoughts, less favorable psychosocial working conditions, decreased positive mental well-being, and higher levels of negative work-home interaction compared to the general population (26). Canadian veterinarians were found to fare worse than the general population in terms of stress, burnout, depression, anxiety, compassion fatigue, suicidal ideation, and resilience. These factors are interconnected; burnout is linked to compassion fatigue (27, 28), and anxiety and depression can lead to both burnout and suicidal ideation (12). In their work, veterinarians must simultaneously meet professional, emotional, and customer service expectations, often with limited resources (29). Social media increasingly contributes to stress and burnout among veterinarians. Publicly shared client complaints, accusations of malpractice, and cyberbullying through online platforms exacerbate already difficult client relationships and emotional exhaustion (30). In addition, social media use negatively affects burnout and secondary traumatic stress in the veterinary profession (31).

The present study aims to examine the occurrence of suicidal thoughts, tendencies, and mental health challenges among veterinarians in Hungary, Finland, Sweden, Germany, and other Northern-European countries including Denmark, Estonia, and Norway. Furthermore, this study seeks to describe work-related stressors and to explore associations between socio-demographic and work-related factors (such as age, gender, number of weekly working hours, job position, and length of annual leave) and psychological distress as well as suicidal thoughts among veterinarians.

## 2 Materials and methods

### 2.1 Survey methodology and questionnaire

The survey was carried out using an online questionnaire, following a comprehensive literature review to identify key issues related to suicide within the veterinary profession. This review formed the basis for the initial draft of the questionnaire, which was subsequently revised several times to ensure coverage of all relevant themes and issues identified in the literature and during discussions between authors. The original questionnaire was developed in Hungarian and subsequently translated into English. The translation process involved consultation among the research team, including one bilingual co-author with native proficiency in both Hungarian and English. Discrepancies were discussed and resolved through consensus. A pilot version was tested among a small group of participants to ensure clarity and conceptual equivalence across both language versions. After finalization of the questionnaire, it was sent to veterinarians in Hungary, Finland, Sweden, Germany, Estonia, Denmark and Norway. The questionnaire was distributed in English in all countries except Hungary, where the Hungarian version was used.

A link to the questionnaire was disseminated along with a brief description, which included an invitation to participate, information about the study’s objectives, and an overview of the survey’s topic areas. Respondents were informed about the estimated completion time, the anonymity of data collection, and the process for evaluating the results.

The questionnaire (Supplementary data) was filled by veterinarians. Participation in the survey was voluntary and anonymous. All participants provided written informed consent before filling the survey. The study was approved by the Scientific and Innovation Committee of the University of Veterinary Medicine Budapest (Approval code: 2025/05/23/1). Participants were recruited through a convenience sampling approach, using professional veterinary networks, closed online platforms and social media channels that are exclusively accessible for veterinarians. This method was chosen to efficiently reach a large and geographically diverse population of practicing veterinarians across multiple European countries, while ensuring that only those actively working in the profession could participate. Inclusion criteria required participants to hold a veterinary degree and to be currently working or to have previously worked in the veterinary field. Exclusion criteria applied to incomplete responses; only respondents who completed all survey questions were included in the final analysis. Accordingly, 26 partially completed questionnaires were excluded from the dataset. In Hungary the questionnaire was shared in the “Állatorvosok-Vets” Facebook group. Finnish and Swedish veterinarians were primarily contacted via email. In Finland the questionnaire was distributed in a Finnish veterinary Facebook group, while in Sweden it was shared with veterinary groups and published in the Swedish Veterinary Federation (SVF) newsletter. In Germany the questionnaire was distributed via veterinary mailing lists. Responses from Estonia, Denmark and Norway were collected through Swedish and Finnish posts. The survey was available online from 19 July 2021 to 23 February 2022 using Google Forms. Data from the survey responses were analyzed using Microsoft Excel (Microsoft Corporation, Redmond, WA, United States).

The questionnaire consisted of 55 questions and was estimated to take 15 min to complete. Of these, 49 were “closed-questions,” 3 were “semi-closed questions” (including 49 single-choice questions and 3 multiple-choice questions) and 3 were “open-ended questions.” Questions 1–8 gathered demographic information about the respondents, including age, gender, marital status, country of birth, population size of their place of residence, country of employment, population size of their workplace, and the veterinary field in which they worked. Questions 9–13 collected information about their professional background, including current job position, years of experience, average weekly working hours, number of colleagues, and average length of annual leave.

Questions 14–19 were a set of yes/no questions designed to assess the mental and physical status of respondents and inquire whether they had known or heard of a veterinarian who had taken their own life. The next section of the questionnaire (questions 20–51) used a 5-point Likert scale to gage the level of agreement or disagreement with various statements, where 1 = strongly disagree, 2 = disagree, 3 = undecided, 4 = agree and 5 = strongly agree. These items were partly adapted by prior studies on mental health and well-being among veterinarians (3, 7, 8, 32, 33). While the instrument was not based on a fully validated questionnaire, internal consistency was assessed using Cronbach’s alpha for item groups, with most scales reaching acceptable reliability levels (*α* > 0.70). The final four questions (questions 52–55) focused on the associations of different stressors with personal life and work activities, as well as the extent to which workplaces are attentive to the mental health of their veterinarians. These questions covered five topics: mental health, unhealthy behavior, client relations, perceptions about the future of the profession and plans of action/aid-seeking.

### 2.2 Statistical analysis

Questions with dichotomous (yes/no) response options were treated as nominal variables and analyzed using descriptive statistics (percentages) and Pearson’s chi-square (χ^2^) tests. Items measured on 5-point Likert scales (ranging from 1 = strongly disagree to 5 = strongly agree) were treated as ordinal variables. For these, means and standard deviations (Mean ± SD) were calculated, and country-level differences were analyzed using Pearson’s chi-square (χ^2^) tests, one-way ANOVA, and significant results were followed up with Tukey HSD *post hoc* tests, including 95% confidence intervals.

The categorical variables were analyzed using Pearson’s chi-square (χ2) test to determine the significance of differences between countries (Hungary, Finland and Sweden, n = 611). Due to the small sample sizes, data from Germany and other Northern European countries were excluded from these analyses. Two-sided tests were used, with row variables representing questions assessing differences between countries and column variables assessing differences between factors associated with suicide. The results of the chi-square test are reported along with the X-squared value, degrees of freedom (df), sample size (n), *p*-value and Cramer V value. Degrees of freedom indicate the size of the table, smaller *p*-values indicate more significant differences between the responses of veterinarians from different countries. The p-value is inversely proportional to the value of the X-square. The Cramer V value, ranging from 0 to 1, indicates the strength of the relationship between the two variables, with values closer to 1 indicating a stronger relationship.

In addition to the Pearson’s chi-square tests, one-way analysis of variance (ANOVA) was applied to examine mean differences across countries for variables measured on Likert scales. The assumption of normality was assessed visually using histograms and Q-Q plots, minor deviations from normality were considered acceptable. ANOVA results are reported with *F* values, degrees of freedom between and within groups and *p*-values. While chi-square tests assess differences in frequency distributions of categorical responses across countries, ANOVA compares the mean scores of continuous or ordinal variables, providing a complementary perspective on national variations. The overall level of statistical significance was set at *p* < 0.05 in both tests.

*Post-hoc* pairwise comparisons were conducted using the Tukey HSD (Honestly Significant Difference) test in cases where ANOVA indicated significant mean differences across countries. This procedure allowed for the identification of specific national differences in response patterns. The results are reported using conventional notation (e.g., HU > FI, *p* < 0.01) to indicate the direction and significance of the differences. To further aid interpretation, 95% confidence intervals (CI) for the mean differences are also presented. These intervals provide an estimate of the precision of the mean differences and allow to assess the potential range within which the true difference lies.

Furthermore, Cronbach’s alpha coefficients were calculated to evaluate the internal consistency and reliability of the Likert-scale items. Cronbach’s alpha values above 0.70 were interpreted as indicating acceptable internal consistency, based on conventional psychometric criteria. These additional analyses were conducted to better assess the psychometric properties of the survey instrument and to strengthen the robustness of the findings. Statistical analyses were performed using R version 4.2.1 (34) and IBM SPSS Statistics Version 25 (IBM, Armonk, NY, United States) (35).

## 3 Results

### 3.1 Socio-demographic characteristics of veterinarians

A total of 724 veterinarians completely filled out the online questionnaire. The majority of respondents were from Hungary (*n* = 236, 32.6%) and Finland (*n* = 218, 30.1%), followed by Sweden (*n* = 157, 21.7%) and Germany (*n* = 77, 10.6%). The remaining respondents were from other Northern-European countries, including Estonia (*n* = 26, 3.6%), Denmark (*n* = 5, 0.7%) and Norway (*n* = 5, 0.7%), collectively representing 5.0% (*n* = 36) of the sample (Table 1).

**Table 1:** Socio-demographic characteristics of the respondents working in Hungary, Finland, Sweden, Germany and other Northern-European countries.

Variable	Category	Total (*n* = 724)	Hungary (*n* = 236)	Finland (*n* = 218)	Sweden (*n* = 157)	Germany (*n* = 77)	Other Northern-European countries (*n* = 36)
Age group	23–34 years	45.4%	47.9%	40.4%	41.4%	53.2%	61.1%
	35–54 years	46.4%	40.3%	51.4%	51.0%	45.5%	38.9%
	Over 54 years	8.1%	11.9%	8.3%	7.6%	1.3%	0.0%
Gender	Male	11.0%	23.4%	3.7%	5.1%	7.9%	5.6%
	Female	89.0%	76.6%	96.3%	94.9%	92.1%	94.4%
Working hours	Weekly <40 h	49.7%	57.1%	53.2%	46.2%	33.3%	30.6%
	Weekly >40 h	50.3%	42.9%	46.8%	53.8%	66.7%	69.4%
Position	Owner/manager position	28.8%	37.7%	28.9%	26.8%	14.7%	11.4%
	Employee/non-managerial position	71.2%	62.3%	71.1%	73.2%	85.3%	88.6%
Holidays	<14 days per year	9.7%	20.8%	5.0%	3.2%	5.2%	2.8%
	14–28 days per year	45.4%	55.9%	28.4%	48.4%	58.4%	38.9%
	>28 days per year	44.9%	23.3%	66.5%	48.4%	36.4%	58.3%

Other Northern-European countries include responses from veterinarians in Estonia, Denmark and Norway.

The largest age group among respondents was 35–54 years (46.4%), followed closely by those aged 23–34 years (45.4%). The over 54 years age group constituted 8.1% of the sample. Female veterinarians made up the majority of respondents, accounting for 89.0%, while male veterinarians comprised 11.0%.

Regarding working hours, approximately half of the respondents worked more than 40 h per week (50.3%), while the other half worked less than 40 h per week (49.7%). The majority worked in an employee/non-managerial position (71.2%), and the other respondents worked in an owner/manager position (28.8%). When it came to annual leave entitlement, nearly half of the respondents reported having between 14 and 28 days of holidays per year (45.4%), closely followed by those with more than 28 days (44.9%). A smaller proportion had fewer than 14 days of holiday per year (9.7%).

In terms of the veterinary field (Table 2), the majority of respondents worked in small animal medicine (69.6%), followed by vets working in mixed animal practice (18.0%), equine medicine (15.9%) and farm animal medicine (13.3%). A smaller proportion of veterinarians worked in food chain safety authorities (10.8%), teaching/research (9.3%), exotic animal medicine (9.0%) and laboratories (3.0%). The “Other” category (3.2%) included professionals working in the pharmaceutical industry, the fish and rabbit sector, shelters, etc. Only one respondent indicated not working within the veterinary profession; however, this individual had worked as a veterinarian in small animal practice for a long period prior to leaving the profession.

**Table 2:** Veterinary fields of the respondents working in Hungary, Finland, Sweden, Germany and other Northern-European countries.

Working field	Total (*n* = 724)	Hungary (*n* = 236)	Finland (*n* = 218)	Sweden (*n* = 157)	Germany (*n* = 77)	Other Northern-European countries (*n* = 36)
Small Animal Medicine	69.6%	78.0%	66.5%	65.6%	57.1%	77.8%
Mixed Practice	18.0%	12.3%	27.5%	18.5%	13.0%	5.6%
Equine Medicine	15.9%	6.4%	17.4%	25.5%	23.4%	11.1%
Farm Animal Medicine	13.4%	10.6%	11.9%	16.6%	15.6%	22.2%
Authorities/State Vet	10.8%	9.3%	16.1%	9.6%	6.5%	2.8%
Teaching/Research	9.3%	6.4%	9.6%	5.7%	15.6%	27.8%
Exotic Animal Medicine	9.0%	14.0%	6.9%	7.0%	5.2%	5.6%
Laboratory	3.0%	3.8%	0.9%	1.3%	6.5%	11.1%
Other	3.2%	4.2%	1.4%	3.2%	6.5%	0.0%

Other Northern-European countries include responses from veterinarians in Estonia, Denmark and Norway.

Note: As multiple responses were allowed, percentages may add up to more than 100%.

### 3.2 Mental health challenges, medication use, and suicide awareness

**Figure 1:**
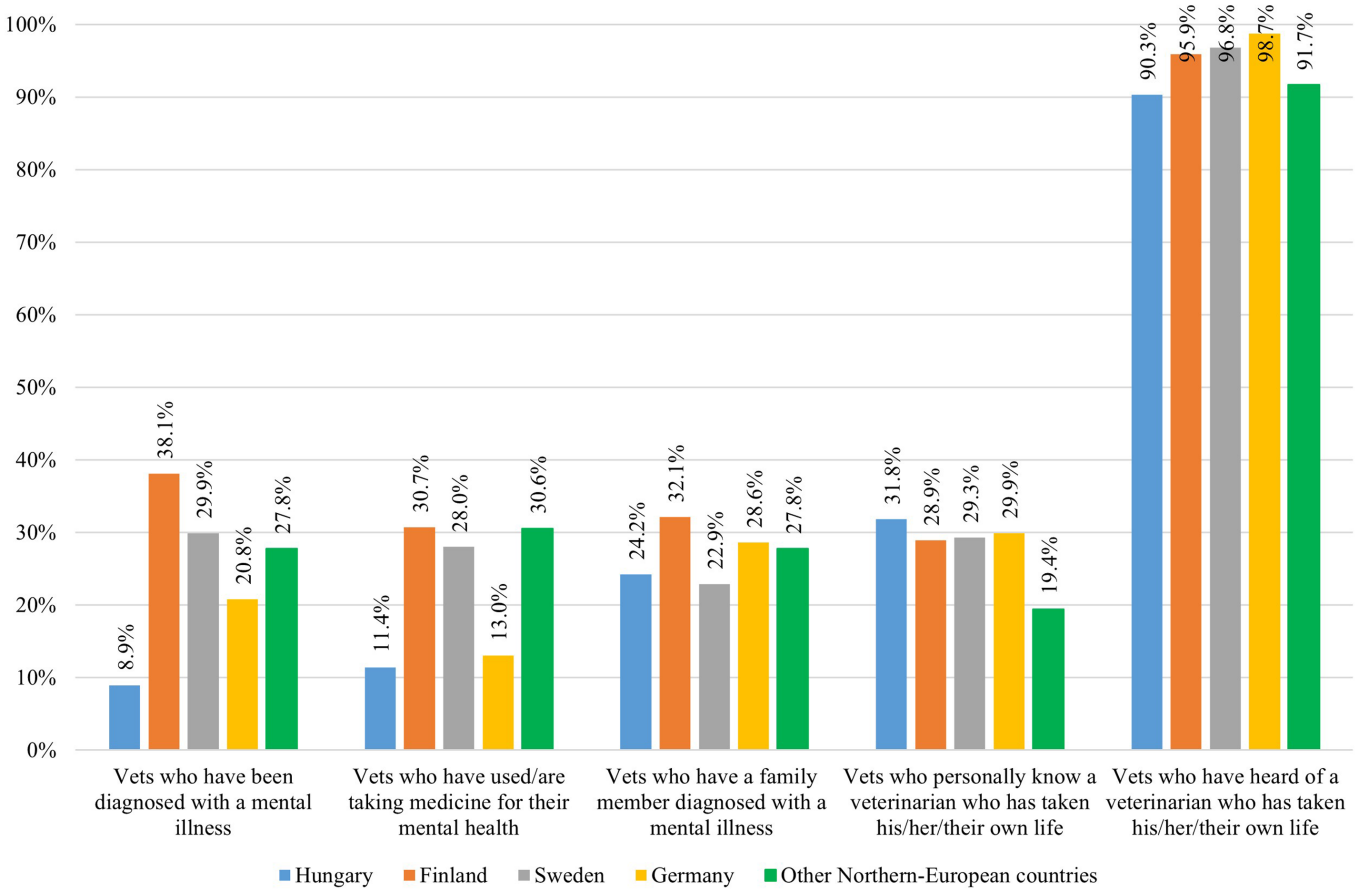
Occurrence of diagnosed mental illness, psychiatric medication use, family history of mental illness and suicide-related awareness among veterinarians from Hungary (*n* = 236), Finland (*n* = 218), Sweden (*n* = 157), Germany (*n* = 77) and other Northern-European countries (*n* = 36).

The occurrence of diagnosed mental illness among veterinarians varied significantly across countries, with an average of 24.4%. Finland reported the highest rate (nearly 40%), while Hungary showed the lowest (less than 10%), representing a significant difference (χ^2^ = 54.940; *p* < 0.0001; Figure 1). Across most countries, younger veterinarians were more likely to be diagnosed with mental illness, except in Hungary, where this was reported by only 7.1% of younger veterinarians. Overall, female veterinarians reported higher rates of mental illness (26.4%) than males (7.8%).

A similar pattern was observed regarding taking medication for mental health issues (overall average: 22.0%), with the highest rate in Finland (nearly 30%) and the lowest in Hungary (11.4%; χ^2^ = 27.701; *p* < 0.0001). Additionally, 26.9% of respondents indicated that a family member had been diagnosed with a mental illness.

Regarding the awareness of suicide, 29.6% of respondents reported personally knowing a veterinarian who had died by suicide. This was more commonly reported by veterinarians over 54 years of age. Male veterinarians were also more likely than female veterinarians to report this experience. Furthermore, 94.3% of the respondents had heard of a veterinarian taking their own life, with all countries reporting rates above 90%.

### 3.3 Perceptions and attitudes toward mental health, suicide, and work-related stress

**Figure 2:**
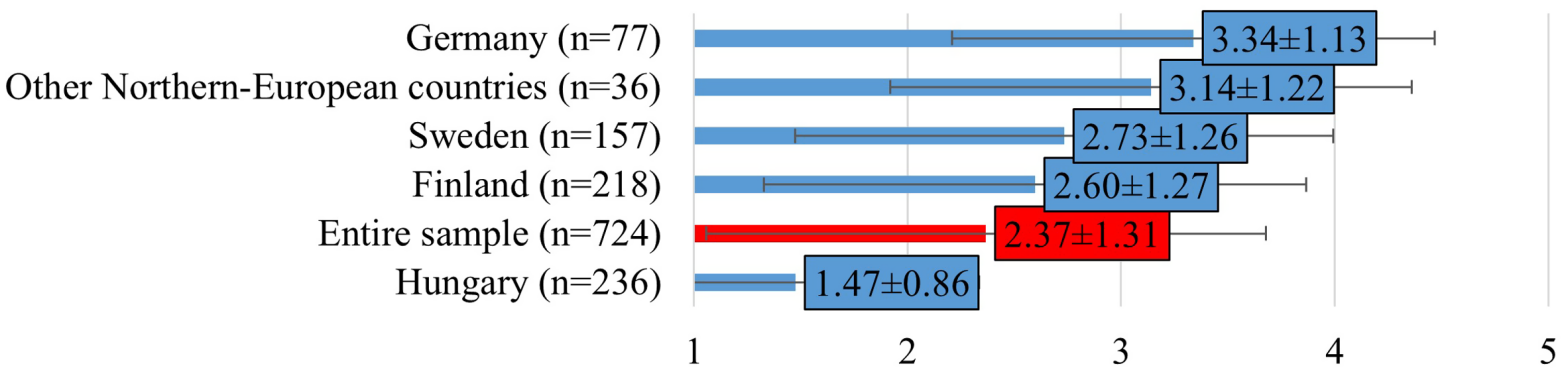
Veterinarians’ perception on whether mental health issues would make them unfit to practice as veterinary professionals (mean ± SD). Responses were given on a 5-point Likert scale, where 1 = strongly disagree, 2 = disagree, 3 = neither agree nor disagree, 4 = agree, 5 = strongly agree. Other Northern-European countries include responses from veterinarians in Estonia, Denmark and Norway.

Most respondents did not consider that mental illness would make them unfit to practice veterinary medicine (mean: 2.37 ± 1.31; Figure 2). However, significant country-level differences were observed (F[2, 608] = 220.774; *p* < 0.0001): Hungarian respondents gave the lowest ratings (mean: 1.47 ± 0.86), while their Swedish (mean: 2.73 ± 1.26) and German (mean: 3.34 ± 1.13) colleagues reported increasingly higher scores.

**Figure 3:**
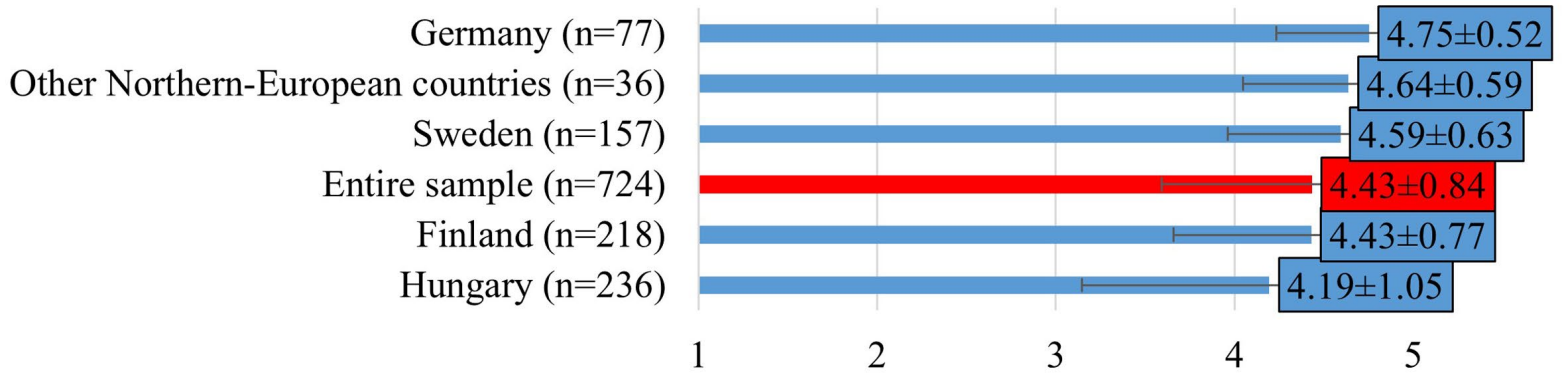
Veterinarians’ perception on whether the suicide rate is higher among veterinarians than in the general population (mean ± SD). Note: Responses were given on a 5-point Likert scale, where 1 = strongly disagree, 2 = disagree, 3 = neither agree nor disagree, 4 = agree, 5 = strongly agree. Other Northern-European countries include responses from veterinarians in Estonia, Denmark and Norway.

Regarding the perceived occurrence of suicide in the veterinary profession, respondents generally believed it to be higher than in the general population (mean: 4.43 ± 0.84; Figure 3), with the highest proportion in Germany (mean: 4.75 ± 0.52) and the lowest in Hungary (mean: 4.19 ± 1.05; F[2, 608] = 10.715; *p* < 0.0001).

The majority of respondents clearly denied ever having seriously considered or attempted suicide (mean: 2.27 ± 1.50), with variations across countries: Hungarian (mean: 2.21 ± 1.52) and German (mean: 1.87 ± 1.34) veterinarians were most likely to deny such thoughts, while Finnish (mean: 2.42 ± 1.56) and Swedish (mean: 2.26 ± 1.49) respondents reported them more frequently.

**Figure 4:**
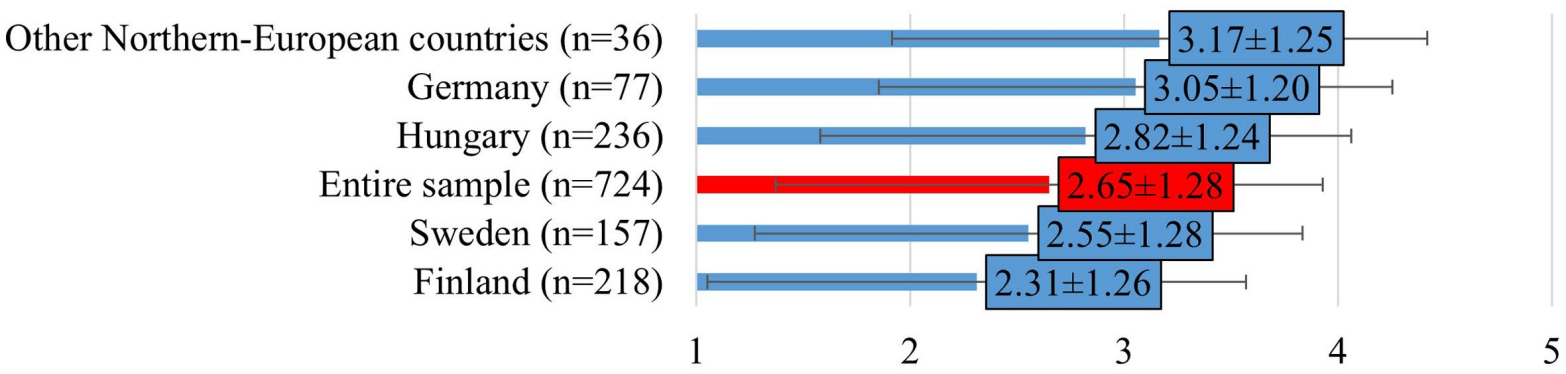
Veterinarians’ perceptions of whether performing euthanasia affects them personally (mean ± SD). Note: Responses were given on a 5-point Likert scale, where 1 = strongly disagree, 2 = disagree, 3 = neither agree nor disagree, 4 = agree, 5 = strongly agree. Other Northern-European countries include responses from veterinarians in Estonia, Denmark and Norway.

The perceived negative impact of work on mental health showed a significant difference (mean: 3.08 ± 1.26), with Swedish (mean: 3.38 ± 1.28) and German (mean: 3.22 ± 1.24) respondents reporting it more frequently than Hungarian (mean: 2.90 ± 1.28) and Finnish (mean: 2.94 ± 1.20) respondents (F[2, 608] = 8.138; *p* < 0.0001). Similarly, emotional burden related to performing euthanasia varied by country (mean: 2.65 ± 1.28), with Germany (mean: 3.05 ± 1.20) reporting the highest emotional impact and Finland the lowest (mean: 2.31 ± 1.26; F[2, 608] = 9.332; *p* < 0.0001; Figure 4).

Finally, the perfectionism and fear of failure were common among veterinarians across all countries (mean: 3.91 ± 1.16), being most pronounced in Hungary (mean: 4.02 ± 1.25) and least reported in Sweden (mean: 3.82 ± 1.18) and Germany (mean: 3.70 ± 1.11; F[2, 608] = 5.031; *p < 0.01*).

### 3.4 Perceptions and attitudes regarding work-life balance, unhealthy habits and perceived workplace support

**Figure 5:**
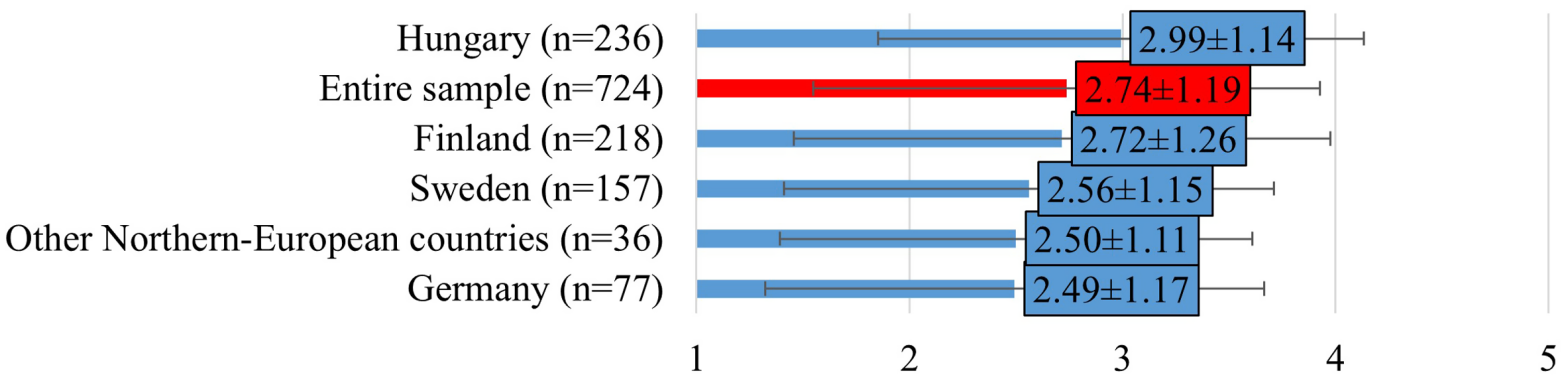
Veterinarians’ perceptions of whether feel they can separate their work and private life (mean ± SD). Responses were given on a 5-point Likert scale, where 1 = strongly disagree, 2 = disagree, 3 = neither agree nor disagree, 4 = agree, 5 = strongly agree. Other Northern-European countries include responses from veterinarians in Estonia, Denmark and Norway.

The ability to separate work and private life varied significantly across countries (mean: 2.74 ± 1.19; F[2, 608] = 79.702; *p* < 0.0001; Figure 5). Respondents from Hungary (mean: 2.99 ± 1.14) and Finland (mean: 2.72 ± 1.26) reported greater difficulty in maintaining work-life balance, while veterinarians in Germany were less affected (mean: 2.49 ± 1.17). On average, respondents tended to agree their work negatively impacted their personal life and contributed to unhealthy habits (mean: 3.34 ± 1.34).

The perception of being forced to work even when ill or during family emergencies was also common (mean: 3.42 ± 1.33), especially in other Northern-European countries (mean: 4.03 ± 1.21) and Finland (mean: 3.60 ± 1.26), while it was less so in Hungary (mean: 2.97 ± 1.36; F[2, 608] = 15.599; *p* < 0.0001).

Social isolation due to long working hours and lack of leisure time emerged as a significant issue (mean: 3.45 ± 1.40), particularly among respondents from Germany (mean: 3.75 ± 1.33), while it was reported less frequently in Finland (mean: 3.19 ± 1.40; F[2, 608] = 3.706; *p < 0.05*).

### 3.5 Perceptions and attitudes on client relations, owner expectations, and communication challenges

There was broad agreement among respondents that clients often expect veterinarians to treat animals free of charge and may prevent necessary interventions for the animal’s benefit (mean: 3.87 ± 1.15). This perception was strong in Germany (mean: 4.36 ± 0.87) and Hungary (mean: 4.04 ± 1.13), while significantly lower in Finland (mean: 3.62 ± 1.16) and Sweden (mean: 3.65 ± 1.20; F[2, 608] = 16.132; *p* < 0.0001).

A strong agreement emerged regarding the perception that animal owners expect an immediate diagnosis (mean: 4.34 ± 0.84), with the highest agreement in Germany (mean: 4.74 ± 0.50) and Hungary (mean: 4.40 ± 0.83), compared to Finland (mean: 4.23 ± 0.85) and Sweden (mean: 4.17 ± 0.90; F[2, 608] = 8.827; *p* < 0.001).

### 3.6 Perceptions of career satisfaction, occupational stress and professional challenges

Most respondents agreed that they had made the right choice by becoming a veterinarian (mean: 3.89 ± 1.09), with the highest proportions in Finland (mean: 4.02 ± 1.00) and Hungary (mean: 3.97 ± 1.08), though the majority in all countries expressed a positive view (F[2, 608] = 10.426; *p* < 0.01).

Moderate agreement was expressed regarding having considered leaving the profession (mean: 2.72 ± 1.37), with the other Northern-European countries (mean: 3.22 ± 1.20) and Sweden showing the highest proportion (mean: 2.97 ± 1.39), and Hungary (mean: 2.62 ± 1.40) and Finland (mean: 2.56 ± 1.33) lower rates (F[2, 608] = 4.735; *p* < 0.01).

There was near-unanimous agreement that the veterinary profession has been under increased stress over the past decade (mean: 4.40 ± 0.86), with the strongest confirmation in Sweden (mean: 4.64 ± 0.65) and the other Northern-European countries (mean: 4.42 ± 0.91). Hungarian respondents also expressed agreement, though somewhat less strongly (mean: 4.25 ± 1.01; F[2, 608] = 17.124; *p* < 0.0001).

### 3.7 Access to mental health support, professional counselling, and self-care skills

The need for professional counseling to cope with work-related stress received a moderate level of agreement among participants (mean: 3.15 ± 1.42), with the highest proportion observed in Finland (3.52 ± 1.31) and the lowest in Hungary (mean: 2.93 ± 1.46) and Germany (mean: 2.74 ± 1.27; F[2, 608] = 10.257; *p* < 0.0001).

Regarding self-help techniques and stress reduction methods, responses were mixed (mean: 3.12 ± 1.18). Swedish (mean: 2.88 ± 1.21) and Hungarian (mean: 3.11 ± 1.23) respondents reported feeling least prepared in this area, while Finnish respondents expressed higher self-confidence (mean: 3.29 ± 1.11; F[2, 608] = 14.933; *p* < 0.0001).

### 3.8 Cross-national comparison of perceptions on mental health and the work environment

Country-level comparisons were conducted using standard statistical tests (mean values ± SD, χ^2^, Cramer’s V, ANOVA including Tukey HSD); reliability was assessed with Cronbach’s alpha (Supplementary Tables 1, 2). The strongest association was found for the statement that mental health problems made respondents feel unfit to be a veterinarian (χ^2^ = 228.3941; *p <* 0.0001; Cramer’s V = 0.486). Significant country-level differences were also observed regarding the negative mental effects of work (χ^2^ = 23.909; *p* < 0.01), the emotional impact of performing euthanasia (χ^2^ = 30.060; *p* < 0.0001), and the association between striving for conscientiousness and feelings of inadequacy or anxiety (χ^2^ = 27.230; *p* < 0.01). Furthermore, veterinarians’ perceptions of unrealistic expectations from clients (χ^2^ = 32.195; *p < 0.0001*), the increased mental stress in the veterinary profession over the past 10 years (χ^2^ = 44.568; *p <* 0.0001) and seeking counseling for work-related stress (χ^2^ = 30.756; *p <* 0.0001) also varied significantly by country. Similarly, the statement *“I feel that I can separate my work and private life”* showed marked international variation (χ^2^ = 157.952; *p <* 0.0001). However, some statements, such as considerations of leaving the profession or the quality of relationships with colleagues, did not show significant differences by country.

In addition to the chi-square analyses, one-way ANOVA tests were performed to compare mean scores across countries for Likert-scale items. The results were consistent with the chi-square findings. The most significant country-level differences were observed in items related to unfit for veterinary profession, separation of work and private life, increased stress in the past 10 years, and owner expectations for free treatments (*p* < 0.0001). Internal consistency of the Likert-scale items was evaluated using Cronbach’s alpha, with most item groups reaching acceptable reliability levels (*α* > 0.70).

### 3.9 Socio-demographic differences in perceptions of mental health, workload, and occupational challenges

Based on the entire sample clear demographic differences were observed in mental health outcomes and work-related perceptions (Supplementary Tables 3, 4). Younger veterinarians (23–34 years old), women, non-managerial staff, those working more than 40 h per week and individuals receiving less than 14 days of annual leave consistently reported higher stress levels, more negative work experiences and greater mental strain.

For example, younger respondents (mean: 4.54 ± 0.72) and women (mean: 4.49 ± 0.75) more frequently perceived suicide occurrence compared to older respondents (aged 54 and above; mean: 3.80 ± 1.17) and men (mean: 3.84 ± 1.25). Similarly, female veterinarians (mean: 3.24 ± 1.38) and non-managerial staff (mean: 3.33 ± 1.34) reported a greater need for counseling and were more likely to experience unhealthy habits due to work-related stress.

Work-related factors also influenced mental health outcomes: veterinarians in non-managerial positions showed higher rates of diagnosed mental illness in Finland (41.4%), Hungary (10.9%), and Sweden (30.4%), while in Germany, practice owners reported higher rates than employees (27.3%). Working long hours and limited annual leave were also associated with greater mental health risks (75.0%).

Regarding career considerations, older participants (mean: 1.92 ± 1.26), men (mean: 2.19 ± 1.30), and those in leadership positions (mean: 2.33 ± 1.31) were more likely to reject the idea of leaving the profession than younger ones aged 23–34 years (mean: 2.91 ± 1.39), women (mean: 2.75 ± 1.37), and veterinary employees (mean: 2.89 ± 1.37). Financial concerns and the perceived declining prestige of the profession were more common among female veterinarians (mean: 4.47 ± 0.79) and employees (mean: 4.49 ± 0.75) than among male respondents (mean: 3.84 ± 1.17) and managers (mean: 4.24 ± 1.02).

Regarding client communications, female veterinarians were more likely to agree that animal owners expect immediate diagnoses (mean: 4.36 ± 0.81) and free treatment purely out of love for animals (mean: 3.90 ± 1.12) than males (means: 4.06 ± 1.00 and 3.52 ± 1.34).

Preparedness for stress management and self-care was notably higher among older (mean: 3.83 ± 1.16), less overworked (mean: 3.21 ± 1.18), and managerial respondents (mean: 3.49 ± 1.13), whereas younger (mean: 2.91 ± 1.13), non-managerial veterinarians (mean: 2.96 ± 1.16), and those working long hours (mean: 3.02 ± 1.17) reported feeling least equipped in this area. Moreover, female veterinarians were more likely to seek professional counseling (mean: 3.24 ± 1.38) compared to men (mean: 2.26 ± 1.33).

## 4 Discussion

### 4.1 Mental distress and suicidal ideation among veterinarians

Our study identified a considerable proportion of veterinarian respondents who had experienced suicidal ideation, with some having seriously considered or attempted suicide. Finnish veterinarian participants reported the highest rates of suicidal ideation or attempts, aligning with broader statistics that show Finland has one of the highest suicide rates globally (15). Across the sample, many participants perceived suicide to be more frequent within the veterinary profession than in the general population, a perception that is consistent with prior findings from the United States (21), the United Kingdoom (36), Canada (12), Australia (13), Finland (16) and Spain (37, 38).

These results underline the need to focus specifically on suicidal thoughts and tendencies as key issues in veterinary mental health research. Our findings mirror international trends: in the US, 25% of veterinarians reported suicidal thoughts, and 5.3% reported serious psychological distress (39). Within our sample, suicidal thoughts were reported more frequently by Finnish and Swedish veterinarian respondents, while Hungarian and German respondents were more likely to deny such experiences, highlighting relevant country-level variation.

Veterinarians’ stress levels and mental well-being also showed clear national differences. According to Jansen et al., the scores of Warwick-Edinburgh Mental Wellbeing Scales (WEMWBS) in 2022/2023 ranged from Hungary’s lowest (23.7) to Sweden’s highest (25.8). Our own data reflected similar regional patterns: Swedish, Finnish and German veterinarians more frequently reported that their work negatively affected their mental health, while Hungarian veterinarians appeared less affected (40).

According to our study, younger veterinarians, female veterinarians, those working more than 40 h per week, individuals with less than 14 days of annual leave, and non-managerial staff reported higher stress levels and greater mental strain. This pattern closely mirrors findings from Germany (5), Portugal (18) and Spain (37, 38), where early-career and female veterinarians similarly reported elevated levels of psychological distress and burnout. Studies from the United States (8) and Canada (12) have also highlighted the compounding effect of excessive working hours and limited time off on veterinarians’ mental health. Furthermore, our observation that the highest rates of diagnosed mental illness were reported among veterinarians aged 23–34 years, particularly women, aligns with international research suggesting that young female professionals are particularly vulnerable to mental health challenges in the veterinary profession (11, 40, 41).

Female veterinarians were also more likely to require counseling for work-related stress, confirming similar international patterns reported in countries such as the United States (8, 10), Canada (12, 42), England and Wales (43) and New Zealand (22). While some studies report no gender differences (5), and others found older or male veterinarians at higher risk (44), our data support the view that younger age, female gender, long working hours and non-managerial positions are key risk factors for psychological distress and suicidal thoughts.

However, these results may be influenced by sample composition, as female respondents in many studies, including ours, were often younger and less experienced, while male participants tended to be older. This imbalance reflects the broader trend of feminization in the veterinary profession (5, 42). In Hungary, for example, more women than men have graduated from the University of Veterinary Medicine Budapest since 2004 (45), and 73.9% of first-year students in the Hungarian-language veterinary program between 2016 and 2020 were female (46).

### 4.2 Work-related stress and psychological risk factors

Our results confirmed that work-related stress is a major concern for veterinarians, consistent with previous research (13). In our sample, a substantial proportion of veterinarian respondents reported experiencing significant anxiety related to their work, particularly due to perfectionism, conscientiousness, and fear of underperformance, like in Spain (9) and Australia (13, 47). These tendencies were most pronounced among veterinarians from Estonia, Denmark, Norway, and Hungary, while Swedish respondents most frequently reported that work negatively affected their mental health. These findings are consistent with international studies from the US (7, 8) and Canada (12), indicating that personality traits such as perfectionism are strongly associated with elevated stress levels and burnout in the veterinary profession. Moreover, Bartram et al. (26) found that UK veterinarians with high personal standards were more prone to anxiety and depressive symptoms. Furthermore, many participants from our study indicated that their professional responsibilities had a negative impact on their private lives and contributed to unhealthy habits. This was particularly evident among those working long hours, holding non-managerial positions, or receiving limited annual leave. These findings are in line with international literature linking excessive workload and poor work-life balance to psychological distress (26, 48).

Regarding perceptions of professional fitness, Hungarian respondents most often disagreed with the notion that mental health problems would render someone unfit to practice, while attitudes in other countries were generally more neutral. This suggests cultural differences in how veterinarians perceive the compatibility of mental health challenges with professional capability, similarly to observations from the United Kingdom (36).

Although our study did not assess factors like neuroticism or student debt directly, work-related stressors observed in our sample, such as long working hours and limited annual leave, are consistent with these known psychological risk factors (8, 10). Therefore, organizational factors appear as key targets for interventions aiming to reduce psychological risk within the veterinary profession.

### 4.3 Work-life balance and unhealthy habits

Our study confirmed that work-life imbalance and unhealthy habits are key contributors to veterinarians’ psychological distress (2, 6, 16). The majority of respondents reported that their work negatively impacted their private life, with the highest rates among those working more than 40 h per week and those with less than 14 days of annual leave. These veterinarians reported significantly higher rates of mental illness, feelings of overwork, isolation, and professional uncertainty compared to those working fewer hours or having longer holidays (49, 50).

Among respondents with limited annual leave, particularly in Germany, reports of diagnosed mental health problems were most common. These findings are in line with international studies highlighting the detrimental effects of inadequate rest and recovery time on mental well-being. Hilbrecht & Smale (42) and Calati et al. (48) emphasized that limited time off and high workloads are significant predictors of psychological distress among veterinarians. This pattern echoes results from studies conducted in the US, which found that excessive working hours contribute to burnout and suicidal ideation (51, 52).

In our sample, German and other Northern European veterinarians most frequently reported being compelled to work even when sick or during family emergencies. These responses suggest that this might remain a major risk factor. Previous research has identified such patterns of social isolation and overwork as being linked to an increased risk of suicidal thoughts and behaviors (36, 53, 54).

Furthermore, studies from other regions have highlighted the broader mental health consequences of such chronic stressors, including compassion fatigue. For example, Spanish veterinarians reported moderate to high levels of compassion fatigue and perceived negative effects of their work on mental health (26), while among Portuguese veterinary professionals, high levels of stress (17.8%), anxiety (27.0%), burnout (27.4%), and compassion fatigue (27.7%) were documented (18). In Hong Kong, 29.4% of veterinarians reported symptoms of depression and anxiety, with secondary traumatic stress increasing suicide risk (14). These findings emphasize that work-life balance issues and unhealthy habits affecting veterinarians’ mental health are not limited to one region but represent a global concern. Our results similarly highlighted the role of long working hours, insufficient leave, and work-related exhaustion as key contributors to psychological distress among veterinarians.

In our study, veterinarians in managerial positions reported lower stress. This contrasts with Pearson et al. study (55), which found that although the difficulties of transitioning into leadership positions improve over time, the pace of work continues to increase, resulting in an undeniable imbalance between work and private life. Moreover, those working fewer than 40 h per week, or with more than 28 days of annual leave reported better work-life balance and lower stress. These findings are consistent with previous studies suggesting that organizational factors, such as workload control (56) and adequate time off (57), act as protective elements against psychological distress. These structural conditions should therefore be prioritized when designing mental health interventions tailored to the veterinary profession, as they can help foster resilience and long-term well-being (49, 58).

### 4.4 Client expectations and communication challenges

Veterinarians face a variety of job stressors that significantly impact their mental well-being, including dealing with clients-related interactions, euthanasia, and managing complex cases. Blair & Hayes (6) and Peixoto (18) found that emotionally demanding client interactions were among the most frequently reported sources of occupational stress. Similarly, research by Ptacek et al. (21) identified euthanasia-related emotional burden and case complexity as key contributors to compassion fatigue and psychological strain in the profession. In our study many respondents, especially those from Germany and Hungary, perceived strong pressure from animal owners to provide an immediate diagnosis. This observation aligns with international literature showing that negative client experiences are directly associated with client-related burnout and, indirectly, with depression (36, 50, 59). In our study, female veterinarians were more likely than their male colleagues to report that unrealistic client expectation contributed to their psychological distress. This observation supports the findings of Rhodes et al. (59), whose structural equation modeling revealed that negative client experiences were directly associated with increased client-related and work-related burnout. While the pathway to depression was indirect, the results emphasize that challenging client interactions can significantly impact veterinarians’ mental health, especially among women.

Dealing with difficult or grieving clients is recognized as a major factor contributing to compassion fatigue (60). Although our study did not directly measure whether such experiences were linked to thoughts of leaving the profession, a considerable proportion of veterinarians, especially in Sweden, reported having considered leaving the field. This tendency mirrors findings from the United States, where a notable share of veterinarians cited client complaints as a key reason for contemplating a career change (58).

Regarding euthanasia (5), overall responses reflected a neutral stance, although country-level differences were evident. Hungarian and German veterinarians reported higher emotional distress related to performing euthanasia, whereas Finnish and Swedish respondents experienced this stress to a lesser extent. This is consistent with Kabboush et al. (61), who found that euthanasia was the least burdensome for Swedish veterinarians.

### 4.5 Career satisfaction and workplace support

Most surveyed veterinarians in Hungary, Finland, and Sweden reported satisfaction with their career choice, although a notable proportion, particularly in Sweden, had considered leaving the profession at some point. This reflects international trends: while US studies show higher dissatisfaction rates (42.6%) (7, 8), our European data present a more positive picture.

Hungarian veterinarians reported greater resilience, and less workplace conflict compared to German and Swedish colleagues, possibly reflecting cultural differences. Despite this, only a minority of respondents across countries felt they had sufficient professional support at their workplace. Although most felt comfortable asking for help, these findings underline the importance of fostering supportive workplace environments. Prior research emphasizes that a safe, open, and supportive workplace culture is one of the strongest predictors of positive mental health outcomes in the veterinary profession (10).

### 4.6 Access to mental health support and help-seeking behavior

Access to mental health services showed marked country-level differences. In our study, Hungarian veterinarians were more likely to perceive a lack of accessible mental health services, while respondents from Finland and Sweden more frequently reported adequate access. These differences reflect varying perceptions of support availability rather than actual usage or awareness. In contrast, a US-based study found that although 83% of veterinarians perceived mental health services as accessible, only 12% had utilized them, including 15.8% of those experiencing serious psychological distress, highlighting a gap between perceived availability and actual engagement with support services (7, 10).

These patterns suggest that even where services exist, awareness and actual utilization remain limited, both in Europe and internationally. Promoting mental health resources more effectively is therefore essential. Prior research emphasizes that workplace culture plays a crucial role: mentoring programs, open communication, and a supportive environment can strengthen mental health outcomes (29, 62). However, individual-level interventions alone, such as mindfulness training, may improve compassion satisfaction but are unlikely to reduce burnout without systemic organizational support. Factors most strongly associated with positive mental health among veterinarians include job satisfaction, good work-life balance, social support, and financial security (63).

### 4.7 Limitations

It is important to note that the findings of the present study are based on data collected through an online questionnaire, which inherently limits responses to individuals who have internet access and are familiar with online surveys. This may have led to potential biases in the sample, reflected in the overrepresentation of younger and female veterinarians, an issue commonly observed in similar survey-based research (5, 42). While statistical methods exist to correct for such imbalances, addressing them falls outside the scope of the present study. Moreover, recruitment strategies differed slightly across countries, which may have affected the engagement and composition of respondents in each country. This variation could have introduced unmeasured sampling biases and should be considered when interpreting cross-national comparisons. Another limitation of our study is that the questionnaire was not based on a fully validated instrument; the item set was partly adapted from previous studies and partly self-developed. This may affect the generalizability of the results.

## 5 Conclusion

This large-scale cross-national survey explored differences and similarities in suicidal ideation, mental health and workplace stress among veterinarians in several European countries including Hungary, Germany, and Scandinavian nations. As research from these regions is limited, the study provides new insights and contributes to a more nuanced understanding of veterinary mental health in Europe. By incorporating data from underrepresented countries such as Hungary and Finland, it expands the geographical scope of existing literature and highlights both shared challenges and country-specific stressors within the profession.

The findings emphasize the need for greater regional awareness and for culturally and systemically adapted mental health resources. Strengthening workplace support systems and improving access to psychological care, especially in countries with limited mental health infrastructure, should be a priority. These results may guide both national associations and pan-European initiatives in tailoring interventions that reflect the realities and needs of veterinary professionals across diverse European contexts.

The most significant differences among the surveyed countries were observed in the perceived impact of euthanasia, the work-life balance, professional support in the workplace, client expectations, perceptions of the veterinary profession’s status and the access to mental health services. Despite these differences, several key similarities emerged across all countries. Notably, most veterinarians reported having heard of or personally known a veterinarian who had died by suicide. However, it is encouraging that the majority of the respondents did not report experiencing suicidal thoughts themselves and expressed a commitment to remaining in the profession.

Although veterinarians face a higher risk of suicide compared to the general population, opinions on the issue differ significantly between the countries surveyed. Hungarian veterinarians were the least likely to acknowledge suicide as a common problem in the profession, while German and Swedish veterinarians were more likely to recognize its occurrence. Further research is needed within the veterinary community to develop targeted strategies for addressing mental distress and suicidal ideation. These efforts should be adapted to national contexts, as noticeable differences were observed in the availability of mental health services. For example, the lack of accessible mental health support for veterinarians in Hungary, may represent a critical factor to consider in the design of future intervention programs.

## Data Availability

The raw data supporting the conclusions of this article will be made available by the authors, without undue reservation.
