# Supplementary material for: A cross-national study on mental health, psychological distress and suicidal ideation among veterinarians in multiple European countries
Source: Front Vet Sci. 2025 Sep 15;12:1634139. doi: 10.3389/fvets.2025.1634139 (PMC12477011; doi:10.3389/fvets.2025.1634139)
Supplement: Supplementary file 1 [file Data_Sheet_1.PDF]

Supplementary Material

1 Supplementary Data

This research is conducted on issues related to mental health in the veterinary profession. Veterinarians have a suicide rate three to four times greater than that of the general population. We are examining the mental health status and propensity to commit suicide of Veterinarians. The questionnaire will be sent out to Veterinarians in a number of other countries. The submitted responses will be compared on a country-by-country basis to gain a comprehensive view of the ideas and prospectives of Veterinarians on this topic.

The questionnaire will be completed in a completely anonymous format; the data will be processed in aggregate form. The questionnaire takes approximately 15 minutes to complete.

Thank you for contributing to our growing understanding of mental health in the veterinary field!

Socio Demographic Questions:

Q 1: Which age group do you belong to?

- 23-34
- 35-44
- 45-54
- 55-64
- 65-74
- Older than 74

Q 2: What is your gender?

- Female
- Male
- I prefer not to say.

Q 3: What is your current marital status?

- Unmarried
- Married
- Divorced
- Widow
- Other: ____________________________

Q 4: Where is your place of residence?[1]

- <10,000
- 10,000-100,000
- 100,001-500,000
- 500,001-1,000,000
- >1,000,000

Q5: Please write the name of the country where you were born!

Q 6: Where is your place of work? (You may choose more than one answer!)[2]

- <10,000
- 10,000-100,000
- 100,001-500,000
- 500,001-1,000,000
- >1,000,000

Q 7: Please write the name of the country where you work!

Q 8: What field are you currently working in within veterinary medicine? (You may choose more than one answer!)

- Small Animal Medicine
- Exotic Animal Medicine
- Farm Animal Medicine
- Equine Medicine
- Mixed Practice
- Laboratory
- Authorities/State Vet
- Teaching/Research
- Other: ____________________

[1] For Hungarian Veterinarians:
- Capital city
- Large town (>100,000)
- Middle-sized town (50,001-100,000)
- Small town (10,000-50,000)
- Other settlement (<10,000)

[2] For Hungarian Veterinarians:
- Capital city
- Large town (>100,000)
- Middle-sized town (50,001-100,000)
- Small town (10,000-50,000)
- Other settlement (<10,000)

Q 9: What position are you in at your current job now?

- Owner / manager position
- Employee / non - managerial position
- Retired
- Other: ___________________________

Q 10: How many years of professional experience do you have?

- Less than 1 year
- 1-3 years
- 4-6 years
- 7-10 years
- 11-20 years
- 21-30 years
- 30 <

Q 11: Which of the following categories most accurately describes your average weekly work hours?

- weekly less than 20 hours
- weekly 20-30 hours
- weekly 31-40 hours
- weekly 41-50 hours
- weekly 51-60 hours
- weekly over 60 hours
- Unemployed, looking for a job.
- Unemployed, not looking for a job.

Q 12: How many colleagues do you work with on a daily basis in your workplace?

- Alone
- 1-5 people
- 6-10 people
- 11-20 people
- More than 20 people.

Q 13: How many holiday/free days are you given a year?

- Less than 14 days
- 14-28 days
- 29-35 days
- 36-42 days
- More than 42 days

Q 14: Have you been diagnosed with a Mental illness? (ex. Depression, Anxiety, Bipolar, Eating Disorder): Yes/No

Q 15: I have used / am taking medicine for my mental health: Yes/No

Q 16: Is anyone in your family diagnosed with a mental illness? Yes/No

Q 17: Have you suffered a physical injury due to your job?

- No
- Yes, but it’s gone away.
- Yes, it’s a permanent injury.

Q 18: I personally know a veterinarian who has committed suicide. Yes / No

Q 19: I have heard of a veterinarian who committed suicide. Yes/No

General Questions:

Likert 1-5 scale: Select on a scale from 1- 5 how much do you agree with the following statements:

1: strongly disagree, 2: disagree, 3: undecided, 4: agree, 5: strongly agree

Q 20: I feel I picked the right profession when I became a veterinarian.

Q 21: I often think about whether or not to leave my veterinary career.

Q 22: My role at work has a negative impact on my mental health.

Q 23: I often feel I have to go to work even when I am sick or have family emergencies because of the nature of my work.

Q 24: I would need professional counseling due to stress at work / I use professional counseling due to stress at work.

Q 25: I have had suicidal thoughts.

Q 26: I have seriously considered or tried to commit suicide because I believe that suicide is the only way out of the problems present in life.

Q 27: My work has caused me to develop unhealthy habits and tendencies in my personal life.

Q 28: The suicide rate is higher among veterinarians than the general population.

Q 29: I feel that performing euthanasia affects me personally.

Q 30: I feel my work is unfulfilling and draining.

Q 31: Younger / recent veterinarians and female veterinarians are more likely to experience more work-related stress and disadvantage than experienced and male veterinarians.

Q 32: The income level and status of the veterinary professions has declined in recent years, and this worries me greatly.

Q 33: Veterinarians have been under increased stress for the past 10 years.

Q 34: Attitudes towards newly trained veterinarians have changed positively in recent years.

Q 35: In my work, I always strive for conscientiousness, accuracy, but I feel that in some cases I am not good enough, so sometimes I get anxious.

Q 36: I often go to my workplace anxiously about what to do on a given day (unexpected cases, time-consuming administrative tasks, interventions that take longer than expected).

Q 37: I often go to my workplace anxiously because of the manager / co-workers (humiliation, competition, too many people working in one place, disagreements, conflicts, tension).

Q 38: If I run into an obstacle while working, I'm afraid to ask for help due to what others might think.

Q 39: I often feel isolated from society due to long working hours; I feel like I don't have enough free time and because of this I have to face the consequences (for example not having family responsibilities, not enough time for sports and hobbies).

Q 40: I often feel like I don't get enough professional support in the workplace.

Q 41: Establishing and maintaining (customer) relationships is very important, but I often encounter barriers to communication.

Q 42: It's important to keep up with technological and professional developments and practical changes within a particular veterinary field, but I feel it's hard to keep up.

Q 43: Animal owners often expect a veterinarian to treat animals free of charge just for the sake of animal love and do not allow certain interventions that would benefit the animal.

Q 44: Animal owners often expect an immediate diagnosis to be made.

Q 45: I feel that I can discuss any personal mental issues with my colleagues/work.

Q 46: My government/country has accessible mental health services I could use in case I needed it.

Q 47: I feel that having mental health issues makes me unfit to be a veterinary professional.

Q 48: I feel that I can separate my work and private life.

Q 49: If I feel myself getting overwhelmed and my mental health deteriorating, I have a plan of action set in place to help myself.

Q 50: I know how to perform self-care and know how to destress myself if life gets overwhelming.

Summary Question:

Likert 1-5 scale: Select on a scale from 1- 5 how much do you agree with the following statements:

1: strongly disagree, 2: disagree, 3: undecided, 4: agree, 5: strongly agree

Q 51: Evaluate the following stressors related to veterinary work and how they affect your veterinary work:

- Burn-out
- Fatigue, emotional exhaustion
- Euthanasia
- Ethical challenges
- Financial difficulties
- Complaints from dissatisfied animal owners
- High expectations of animal owners
- Work-related cyberbullying
- Fulfillment of requirements in practice
- Practice management business, not enough profit

- Fear of making mistakes in the profession
- Daily contact with staff
- Professional competition
- Fear of malpractice litigation
- There is little social support
- Lack of participation in decision - making

Q 52: Evaluate the following stressors related to veterinary work, and how they affect your mental health in your private life:

- Burn-out
- Fatigue, emotional exhaustion
- Euthanasia
- Ethical challenges
- Financial difficulties
- Complaints from dissatisfied animal owners
- High expectations of animal owners
- Work-related cyberbullying
- Fulfillment of requirements in practice
- Practice management business, not enough profit
- Fear of making mistakes in the profession
- Daily contact with staff
- Professional competition
- Fear of malpractice litigation
- There is little social support
- Lack of participation in decision - making

Q 53: How does your work regard mental health? (You may choose more than one answer!)

- They allow us mental health days
- They do mental health awareness discussions
- They offer counseling
- Nothing

Q 54: If you have any additional comments regarding anything mentioned in the questionnaire, please add them below: ____
