# Supplementary material for: A cross-national study on mental health, psychological distress and suicidal ideation among veterinarians in multiple European countries
Source: Front Vet Sci. 2025 Sep 15;12:1634139. doi: 10.3389/fvets.2025.1634139 (PMC12477011; doi:10.3389/fvets.2025.1634139)
Supplement: Supplementary file 2 [file Data_Sheet_2.PDF]

frontiers

Supplementary Material

1 Supplementary Tables

Supplementary Table 1. Associations between selected mental health-related statements and veterinarians' country of origin (n=611).

Statements (n=31)	Pearson's chi-square (χ2) test			Cramer's V value	ANOVA					Cronbach's α
	X-squared	df	p value		F	df_between, df_within groups	p value	Tukey HSD	95% CI	
Have you been diagnosed with a mental illness?	54.940	2	$p<0.0001$*	0.300						
Have you used/are taking medicine for your mental health?	27.701	2	$p<0.0001$*	0.213						
Is anyone in your family diagnosed with a mental illness?	5.186	2	$p=0.075$	0.092						
I personally know a veterinarian who has committed suicide.	0.514	2	$p=0.774$	0.029						
I have heard of a veterinarian who committed suicide.	9.347	2	$p<0.01$*	0.124						
The suicide rate is higher among veterinarians than the general population.	25.689	8	$p<0.01$*	0.145	10.715	2, 608	$p<0.0001$*	HU < FI ($p<0.05$*), HU < SE ($p<0.0001$*), FI < SE ($p=0.171$)	(4.31–4.45)	0.746
I have had suicidal thoughts.	4.877	8	$p=0.771$	0.063	1.087	2, 608	$p=0.338$	HU < FI ($p=0.325$), HU < SE ($p=0.947$), FI > SE ($p=0.591$)	(2.18–2.42)	0.739
I have seriously considered or tried to commit suicide because I believe that suicide is the only way out of the problems present in life.	19.006	8	$p<0.05$*	0.125	2.248	2, 608	$p=0.107$	HU < FI ($p=0.149$), HU < SE ($p=0.198$), FI = SE ($p=1.000$)	(1.46–1.64)	0.745
I feel that having mental health issues makes me unfit to be a veterinary professional.	288.941	8	$p<0.0001$*	0.486	220.774	2, 608	$p<0.0001$*	HU < FI ($p<0.0001$*), HU < SE ($p<0.0001$*), FI < SE ($p=0.823$)	(2.67–2.89)	0.766
My role at work has a negative impact on my mental health.	23.909	8	$p<0.01$*	0.140	8.138	2, 608	$p<0.0001$*	HU < FI ($p=0.946$), HU < SE ($p<0.01$*), FI < SE ($p<0.01$*)	(2.94–3.14)	0.726

I feel that performing euthanasia affects me personally.	30.060	8	*p<0.0001**	0.157	9.332	2, 608	*p<0.0001**	HU > FI (*p<0.0001**), HU > SE (p=0.098), FI < SE (p=0.156)	(2.47–2.67)	0.750
In my work, I always strive for conscientiousness, accuracy, but I feel that in some cases I am not good enough, so sometimes I get anxious.	27.230	8	*p<0.01**	0.149	5.031	2, 608	*p<0.01**	HU < FI (p=0.263), HU > SE (p=0.171), FI > SE (*p<0.01**)	(2.71–2.87)	0.760
I often go to my workplace anxiously about what to do on a given day (unexpected cases, time-consuming administrative tasks, interventions that take longer than expected).	25.471	8	*p<0.01**	0.144	1.436	2, 608	p=0.239	HU > FI (p=0.480), HU < SE (p=0.238), FI > SE (p=0.843)	(3.83–4.01)	0.733
I often go to my workplace anxiously because of the manager / co-workers (humiliation, competition, too many people working in one place, disagreements, conflicts, tension).	9.849	8	p=0.276	0.090	0.258	2, 608	p=0.773	HU > FI (p=0.760), HU > SE (p=0.980), FI < SE (p=0.896)	(3.32–3.53)	0.726
My work has caused me to develop unhealthy habits and tendencies in my personal life.	11.032	8	p=0.200	0.095	2.572	2, 608	p=0.077	HU < FI (p=0.213), HU < SE (p=0.090), FI < SE (p=0.840)	(3.17–3.38)	0.730
I feel that I can separate my work and private life.	157.952	8	*p<0.0001**	0.360	79.702	2, 608	*p<0.0001**	HU < FI (*p<0.0001**), HU < SE (*p<0.0001**), FI < SE (p=0.482)	(2.10–2.30)	0.747
I often feel I have to go to work even when I am sick or have family emergencies because of the nature of my work.	36.979	8	*p<0.0001**	0.246	15.599	2, 608	*p<0.0001**	HU < FI (*p<0.0001**), HU < SE (*p<0.0001**), FI > SE (p=0.849)	(3.23–3.44)	0.741
I often feel isolated from society due to long working hours; I feel like I don't have enough free time and because of this I have to face the consequences (for example not having family responsibilities, not enough time for sports and hobbies).	26.521	8	*p<0.01**	0.147	3.706	2, 608	*p<0.05**	HU > FI (p=0.712), HU > SE (*p<0.05**), FI > SE (p=0.129)	(1.78–1.96)	0.739
If I run into an obstacle while working, I'm afraid to ask for help due to what others might think.	18.817	8	*p<0.05**	0.124	2.543	2, 608	p=0.079	HU > FI (p=0.426), HU > SE (p=0.066), FI > SE (p=0.527)	(2.18–2.40)	0.732
I often feel like I don't get enough professional support in the workplace.	13.850	8	p=0.086	0.106	3.978	2, 608	*p<0.05**	HU > FI (*p<0.05**), HU > SE (p=0.584), FI < SE (p=0.270)	(3.28–3.51)	0.735
Establishing and maintaining (customer) relationships is very important, but I often encounter barriers to communication.	25.846	8	*p<0.01**	0.145	1.869	2, 608	p=0.155	HU < FI (p=0.479), HU < SE (p=0.140), FI < SE (p=0.270)	(2.63–2.84)	0.736

Animal owners often expect an immediate diagnosis to be made.	32.195	8	*p<0.0001**	0.163	8.827	2, 608	*p<0.0001**	HU > FI (*p<0.0001**), HU < SE (*p<0.01**), FI < SE (p=0.972)	(3.70–3.88)	0.742
Animal owners often expect a veterinarian to treat animals free of charge just for the sake of animal love and do not allow certain interventions that would benefit the animal.	37.945	8	*p<0.0001**	0.176	16.132	2, 608	*p<0.0001**	HU < FI (*p<0.0001**), HU < SE (*p<0.0001**), FI < SE (p=0.454)	(3.41–3.59)	0.739
I feel I picked the right profession when I became a veterinarian.	26.719	8	*p<0.01**	0.148	10.426	2, 608	*p<0.0001**	HU < FI (p=0.841), HU > SE (*p<0.0001**), FI > SE (*p<0.0001**)	(3.79–3.96)	0.779
I often think about whether or not to leave my veterinary career.	11.913	8	p=0.155	0.099	4.735	2, 608	*p<0.01**	HU > FI (p=0.858), HU > SE (*p<0.05**), FI < SE (*0<0.05**)	(2.58–2.80)	0.735
Veterinarians have been under increased stress for the past 10 years.	44.568	8	*p<0.0001**	0.191	17.124	2, 608	*p<0.0001**	HU < FI (p=0.684), HU < SE (*p<0.0001**), FI < SE (*p<0.0001**)	(3.78–3.96)	0.744
The income level and status of the veterinary professions has declined in recent years, and this worries me greatly.	29.727	8	*p<0.0001**	0.156	0.287	2, 608	p=0.750	HU > FI (p=0.935), HU > SE (p=0.729), FI > SE (p=0.903)	(3.65–3.83)	0.745
It's important to keep up with technological and professional developments and practical changes within a particular veterinary field, but I feel it's hard to keep up.	13.898	8	p=0.084	0.107	2.437	2, 608	p=0.088	HU < FI (p=0.973), HU < SE (p=0.097), FI < SE (p=0.158)	(2.56–2.74)	0.736
I would need professional counseling due to stress at work / I use professional counseling due to stress at work.	30.756	8	*p<0.0001**	0.159	10.257	2, 608	*p<0.0001**	HU < FI (*p<0.0001**), HU < SE (p=0.376), FI < SE (*p<0.05**)	(3.07–3.30)	0.725
My government/country has accessible mental health services I could use in case I needed it.	9.700	8	p=0.287	0.089	1.751	2, 608	p=0.174	HU > FI (p=0.153), HU > SE (p=0.823), FI < SE (p=0.526)	(2.87–3.07)	0.775
I know how to perform self-care and know how to destress myself if life gets overwhelming.	32.177	8	*p<0.0001**	0.162	14.933	2, 608	*p<0.0001**	HU > FI (*p<0.01**), HU > SE (*p<0.0001**), FI > SE (p=0.146)	(2.85–3.05)	0.767

An asterisk (*) denotes a statistically significant association (p ≤ 0.05). Items 1–5 were dichotomous (yes/no) questions; therefore, ANOVA and Cronbach's alpha were not applicable and have been omitted. Country abbreviations used in Tukey HSD post hoc results: HU = Hungary, FI = Finland, SE = Sweden.

Supplementary Table 2. Country-level comparison of veterinarians' opinions on selected mental health-related statements (mean values ± SD; n=724).

**Statements (n=26)**	**Total sample (n=724)**	**Hungary (n=236)**	**Finland (n=218)**	**Sweden (n=157)**	**Germany (n=77)**	**Other Northern-European countries (n=36)**
The suicide rate is higher among veterinarians than the general population.	4.43±0.84	4.19±1.05	4.43±0.77	4.59±0.63	4.75±0.52	4.64±0.59
I have had suicidal thoughts.	2.27±1.50	2.21±1.52	2.42±1.56	2.26±1.49	1.87±1.34	2.58±1.25
I have seriously considered or attempted suicide because I believe that suicide is the only way out of the problems present in my life.	1.52±1.08	1.43±1.05	1.63±1.17	1.63±1.15	1.27±0.82	1.39±0.77
I feel that having mental health issues would make me unfit to be a veterinary professional.	2.37±1.31	1.47±0.86	2.60±1.27	2.73±1.26	3.34±1.13	3.14±1.22
My role at work has a negative impact on my mental health.	3.08±1.26	2.90±1.28	2.94±1.20	3.38±1.28	3.22±1.24	3.56±1.08
I feel that performing euthanasia affects me personally.	2.65±1.28	2.82±1.24	2.31±1.26	2.55±1.28	3.05±1.20	3.17±1.25
In my work, I always strive for conscientiousness, accuracy, but I feel that in some cases I am not performing well enough, so I get anxious.	3.91±1.16	4.02±1.25	3.89±1.07	3.82±1.18	3.70±1.11	4.19±1.01
I often go to my workplace anxious about daily tasks (unexpected cases, time-consuming administrative tasks, interventions that take longer than expected).	3.43±1.35	3.47±1.36	3.38±1.34	3.44±1.37	3.29±1.41	3.86±1.07
I often go to my workplace anxiously because of the manager / co-workers (humiliation, competition, too many people working in one place, disagreements, conflicts, tension).	2.30±1.37	2.43±1.50	2.27±1.28	2.11±1.32	2.18±1.28	2.72±1.30
My work has caused me to develop unhealthy habits and tendencies in my personal life.	3.34±1.34	3.12±1.39	3.33±1.29	3.41±1.34	3.73±1.15	3.67±1.41
I feel that I can separate my work and private life.	2.74±1.19	2.99±1.14	2.72±1.26	2.56±1.15	2.49±1.17	2.50±1.11
I often feel I must go to work even when I am sick or have family emergencies because of the nature of my work.	3.42±1.33	2.97±1.36	3.60±1.26	3.52±1.25	2.82±1.25	4.03±1.21
I often feel isolated from society due to long work hours; I feel I do not have enough free time and because of this I have to face the consequences (for example not having family time, not enough time for sports and hobbies).	3.45±1.40	3.56±1.39	3.19±1.40	3.42±1.43	3.75±1.33	3.72±1.23
If I run into an obstacle while working, I am afraid to ask for help due to what others might think.	1.92±1.16	1.98±1.28	1.89±1.16	1.66±087	2.17±1.19	2.22±1.15
I often feel like I do not get enough professional support in the workplace.	2.80±1.35	2.61±1.44	2.76±1.32	2.88±1.27	3.14±1.25	3.17±1.25
Establishing and maintaining (customer) relationships is very important, but I often encounter barriers in communication.	2.71±1.18	2.58±1.20	2.60±1.13	2.83±1.22	3.08±1.17	3.03±1.08
Animal owners often expect an immediate diagnosis to be made.	4.34±0.84	4.40±0.83	4.23±0.85	4.17±0.90	4.74±0.50	4.36±0.87
Animal owners often expect a veterinarian to treat animals free of charge just for the sake of animal love and do not allow certain interventions that would benefit the animal.	3.87±1.15	4.04±1.13	3.62±1.16	3.65±1.20	4.36±0.87	4.14±1.02
I feel I picked the right profession when I became a veterinarian.	3.89±1.09	3.97±1.08	4.02±1.00	3.54±1.18	3.97±1.12	3.94±0.89
I often consider if I should leave my veterinary career.	2.72±1.37	2.62±1.40	2.56±1.33	2.97±1.39	2.75±1.39	3.22±1.20

Veterinarians have been under increased stress for the past 10 years.	4.40±0.86	4.25±1.01	4.41±0.80	4.64±0.65	4.39±0.80	4.42±0.91
The income level and status of the veterinary professions has declined in recent years, and this worries me greatly.	3.89±1.13	3.67±1.27	3.76±1.09	4.32±0.93	3.88±1.08	4.25±0.87
It’s important to keep up with technological and professional developments and practical changes within a particular veterinary field, but I feel it is hard to keep up.	3.50±1.16	3.18±1.24	3.64±1.08	3.78±1.00	3.48±1.19	3.58±1.27
I would need professional counselling due to stress at work/I use professional counselling due to stress at work.	3.15±1.42	2.93±1.46	3.52±1.31	3.12±1.46	2.74±1.27	3.36±1.46
My government/country has accessible mental health services I could use in case I needed it.	2.79±1.36	1.66±0.98	3.46±1.06	3.53±1.14	2.75±1.22	3.06±1.35
I know how to perform self-care and know how to destress myself if life gets overwhelming.	3.12±1.18	3.11±1.23	3.29±1.11	2.88±1.21	3.13±1.09	3.14±1.20

Note: Responses were given on a 5-point Likert scale, where 1 = strongly disagree and 5 = strongly agree.
Other Northern-European countries include responses from veterinarians in Estonia, Denmark and Norway.

Supplementary Table 3. Veterinarians' opinions on selected mental health-related statements by age and gender (mean values ± SD).

Statements (n=26)	Age group			Gender	
	23-34 years (n=307)	35-54 years (n=322)	Over 54 years (n=59)	Male (n=77)	Female (n=606)
The suicide rate is higher among veterinarians than the general population.	4.54±0.72	4.42±0.84	3.80±1.17	3.84±1.25	4.49±0.75
I have had suicidal thoughts.	2.55±1.62	2.07±1.40	1.66±1.15	1.92±1.29	2.29±1.54
I have seriously considered or attempted suicide because I believe that suicide is the only way out of the problems present in my life.	1.65±1.20	1.44±1.02	1.27±0.76	1.35±0.85	1.54±1.12
I feel that having mental health issues would make me unfit to be a veterinary professional.	2.39±1.31	2.31±1.30	2.08±1.22	1.79±1.22	2.40±1.30
My role at work has a negative impact on my mental health.	3.25±1.17	3.00±1.30	2.36±1.30	2.69±1.27	3.11±1.26
I feel that performing euthanasia affects me personally.	2.64±1.25	2.63±1.28	2.51±1.36	2.38±1.21	2.65±1.28
In my work, I always strive for conscientiousness, accuracy, but I feel that in some cases I am not performing well enough, so I get anxious.	4.17±1.05	3.76±1.18	3.24±1.32	3.14±1.36	3.99±1.11
I often go to my workplace anxious about daily tasks (unexpected cases, time-consuming administrative tasks, interventions that take longer than expected).	3.64±1.33	3.31±1.33	2.76±1.41	2.87±1.39	3.47±1.34
I often go to my workplace anxiously because of the manager / co-workers (humiliation, competition, too many people working in one place, disagreements, conflicts, tension).	2.36±1.40	2.25±1.33	2.05±1.44	2.08±1.31	2.31±1.38
My work has caused me to develop unhealthy habits and tendencies in my personal life.	3.46±1.27	3.35±1.30	2.46±1.52	3.05±1.38	3.35±1.33
I feel that I can separate my work and private life.	2.69±1.17	2.76±1.19	2.98±1.37	2.97±1.20	2.73±1.20
I often feel I must go to work even when I am sick or have family emergencies because of the nature of my work.	3.35±1.34	3.47±1.30	3.17±1.42	3.12±1.24	3.42±1.34
I often feel isolated from society due to long work hours; I feel I do not have enough free time and because of this I have to face the consequences (for example not having family time, not enough time for sports and hobbies).	3.67±1.32	3.35±1.41	2.64±1.55	2.97±1.50	3.49±1.39
If I run into an obstacle while working, I am afraid to ask for help due to what others might think.	2.05±1.23	1.78±1.08	1.78±1.10	1.66±1.02	1.93±1.16
I often feel like I do not get enough professional support in the workplace.	2.88±1.32	2.75±1.36	2.44±1.39	2.56±1.30	2.81±1.35
Establishing and maintaining (customer) relationships is very important, but I often encounter barriers in communication.	2.81±1.20	2.69±1.16	2.17±1.16	2.38±1,17	2.73±1.18
Animal owners often expect an immediate diagnosis to be made.	4.47±0.74	4.31±0.82	3.74±1.10	4.06±1.00	4.36±0.81
Animal owners often expect a veterinarian to treat animals free of charge just for the sake of animal love and do not allow certain interventions that would benefit the animal.	4.05±1.09	3.74±1.18	3.45±1.20	3.52±1.34	3.90±1.12
I feel I picked the right profession when I became a veterinarian.	3.72±1.13	3.94±1.08	4.47±0.75	4.21±0.96	3.85±1.10
I often consider if I should leave my veterinary career.	2.91±1.39	2.64±1.33	1.92±1.26	2.19±1.30	2.75±1.37

Veterinarians have been under increased stress for the past 10 years.	4.49±0.73	4.41±0.89	3.92±1.16	3.84±1.17	4.47±0.79
The income level and status of the veterinary professions has declined in recent years, and this worries me greatly.	3.94±1.12	3.87±1.14	3.51±1.21	3.40±1.38	3.93±.1.10
It's important to keep up with technological and professional developments and practical changes within a particular veterinary field, but I feel it is hard to keep up.	3.42±1.22	3.59±1.06	3.41±1.25	2.99±1.16	3.56±1.14
I would need professional counselling due to stress at work/I use professional counselling due to stress at work.	3.36±1.35	3.06±1.40	2.46±1.59	2.26±1.33	3.24±1.38
My government/country has accessible mental health services I could use in case I needed it.	2.62±1.30	2.87±1.38	3.14±1.43	2.44±1.39	2.83±1.35
I know how to perform self-care and know how to destress myself if life gets overwhelming.	2.91±1.13	3.18±1.18	3.83±1.16	3.27±1.26	3.09±1.17

Note: Responses were given on a 5-point Likert scale, where 1 = strongly disagree and 5 = strongly agree.

Supplementary Table 4. Veterinarians' opinions on selected mental health-related statements by working hours, position and holidays (mean values ± SD).

Statements (n=26)	Working hours		Position		Holidays		
	Weekly <40 hours (n=345)	Weekly >40 hours (n=335)	Owner/manager position (n=194)	Employee/non-managerial position (n=458)	<14 days per year (n=69)	14-28 days per years (n=315)	>28 days per year (n=304)
The suicide rate is higher among veterinarians than the general population.	4.39±0.87	4.46±0.82	4.28±0.98	4.50±0.76	4.45±0.90	4.42±0.84	4.42±0.84
I have had suicidal thoughts.	2.23±1.52	2.26±1.50	1.84±1.31	2.41±1.55	2.45±1.60	2.31±1.53	2.14±1.47
I have seriously considered or attempted suicide because I believe that suicide is the only way out of the problems present in my life.	1.52±1.15	1.51±1.02	1.30±0.84	1.57±1.13	1.71±1.23	1.53±1.11	1.47±1.05
I feel that having mental health issues would make me unfit to be a veterinary professional.	2.23±1.28	2.43±1.32	2.06±1.29	2.48±1.28	1.90±1.20	2.35±1.33	2.39±1.28
My role at work has a negative impact on my mental health.	2.92±1.25	3.19±1.26	2.71±1.26	3.21±1.24	3.38±1.27	3.12±1.24	2.92±1.27
I feel that performing euthanasia affects me personally.	2.57±1.29	2.68±1.26	2.46±1.22	2.70±1.27	2.94±1.22	2.68±1.25	2.49±1.29
In my work, I always strive for conscientiousness, accuracy, but I feel that in some cases I am not performing well enough, so I get anxious.	3.99±1.13	3.78±1.20	3.37±1.29	4.13±1.01	4.12±1.17	4.05±1.08	3.68±1.22
I often go to my workplace anxious about daily tasks (unexpected cases, time-consuming administrative tasks, interventions that take longer than expected).	3.36±1.38	3.46±1.35	2.94±1.38	3.65±1.28	3.83±1.29	3.55±1.29	3.17±1.40
I often go to my workplace anxiously because of the manager / co-workers (humiliation, competition, too many people working in one place, disagreements, conflicts, tension).	2.20±1.36	2.36±1.37	1.83±1.15	2.50±1.42	2.45±1.49	2.38±1.39	2.14±1.32
My work has caused me to develop unhealthy habits and tendencies in my personal life.	3.05±1.35	3.61±1.25	3.02±1.36	3.47±1.29	3.65±1.34	3.40±1.30	3.17±1.35
I feel that I can separate my work and private life.	3.00±1.16	2.50±1.18	2.90±1.22	2.69±1.18	2.57±1.18	2.71±1.14	2.83±1.25
I often feel I must go to work even when I am sick or have family emergencies because of the nature of my work.	3.16±1.35	3.62±1.26	3.38±1.35	3.41±1.32	3.67±1.32	3.41±1.30	3.30±1.35
I often feel isolated from society due to long work hours; I feel I do not have enough free time and because of this I have to face the consequences (for example not having family time, not enough time for sports and hobbies).	2.93±1.39	3.96±1.23	3.11±1.51	3.59±1.33	3.94±1.29	3.63±1.36	3.11±1.41
If I run into an obstacle while working, I am afraid to ask for help due to what others might think.	1.87±1.19	1.91±1.11	1.63±1.03	2.03±1.19	2.17±1.29	2.01±1.21	1.73±1.04

I often feel like I do not get enough professional support in the workplace.	2.56±1.35	2.97±1.30	2.36±1.30	2.95±1.33	2.99±1.50	2.80±1.36	2.71±1.30
Establishing and maintaining (customer) relationships is very important, but I often encounter barriers in communication.	2.70±1.20	2.69±1.17	2.36±1.14	2.86±1.19	2.86±1.09	2.86±1.22	2.49±1.15
Animal owners often expect an immediate diagnosis to be made.	4.33±0.82	4.34±0.86	4.13±1.00	4.42±0.74	4.39±0.79	4.44±0.80	4.21±0.87
Animal owners often expect a veterinarian to treat animals free of charge just for the sake of animal love and do not allow certain interventions that would benefit the animal.	3.83±1.14	3.87±1.18	3.61±1.25	3.96±1.09	4.04±1.17	4.02±1.06	3.64±1.21
I feel I picked the right profession when I became a veterinarian.	3.90±1.09	3.88±1.11	4.24±0.92	3.71±1.14	3.74±1.20	3.79±1.12	4.03±1.03
I often consider if I should leave my veterinary career.	2.66±1.36	2.73±1.39	2.33±1.31	2.89±1.37	2.80±1.45	2.81±1.37	2.55±1.36
Veterinarians have been under increased stress for the past 10 years.	4.41±0.86	4.41±0.87	4.24±1.02	4.49±0.75	4.41±0.79	4.43±0.86	4.38±0.88
The income level and status of the veterinary professions has declined in recent years, and this worries me greatly.	3.89±1.11	3.85±1.18	3.61±1.26	3.99±1.07	3.67±1.22	4.01±1.11	3.78±1.15
It’s important to keep up with technological and professional developments and practical changes within a particular veterinary field, but I feel it is hard to keep up.	3.46±1.18	3.55±1.12	3.36±1.12	3.55±1.16	3.59±1.28	3.47±1.17	3.50±1.12
I would need professional counselling due to stress at work/I use professional counselling due to stress at work.	3.09±1.44	3.18±1.39	2.72±1.46	3.33±1.34	3.43±1.32	3.20±1.40	3.01±1.44
My government/country has accessible mental health services I could use in case I needed it.	2.75±1.39	2.84±1.32	2.71±1.42	2.81±1.34	1.77±0.96	2.50±1.32	3.30±1.26
I know how to perform self-care and know how to destress myself if life gets overwhelming.	3.21±1.18	3.02±1.17	3.49±1.13	2.96±1.16	2.75±1.22	3.02±1.15	3.30±1.18

Note: Responses were given on a 5-point Likert scale, where 1 = strongly disagree and 5 = strongly agree.
